# The mechanism exploration of the non‐colonic toxicity and obesity inhibition of food‐grade κ‐carrageenan by transcriptome

**DOI:** 10.1002/fsn3.2581

**Published:** 2021-09-28

**Authors:** Hui Zhang, Wanxiu Cao, Fang Liu, Yuan Gao, Yaoguang Chang, Changhu Xue, Qingjuan Tang

**Affiliations:** ^1^ College of Food Science and Engineering Ocean University of China Qingdao China; ^2^ Laboratory of Marine Drugs and Biological Products Pilot National Laboratory for Marine Science and Technology Qingdao China

**Keywords:** anti‐obesity, carrageenan metabolism, colitis, degradation, fat accumulation, food‐grade κ‐carrageenan, inflammatory gene, lipid metabolism, metabolic syndrome, transcriptome

## Abstract

Previous study has suggested the colonic nontoxicity and obesity inhibition of food‐grade κ‐carrageenan in obese mice. Further study using transcriptome is important to provide further understanding on the gene expressions of inflammation and obesity. Here, the obese mice without any treatment (HFD) or with 5% food‐grade κ‐carrageenan diet intervention (H5%) were used to perform colonic transcriptome sequencing. The results showed that genes involved in the inflammatory pathways or tight junction protein encoding were not significantly dysregulated by 5% carrageenan. However, the expression of lipid metabolism genes meaningfully changed as evidenced by the decreased gene levels of adipocytokines, lipogenesis, lipid absorption and transport, and the increased adipolysis and oxidation. In addition, the carrageenan metabolism experiments by toluidine blue (TB) staining of colon and high‐performance size exclusion chromatography (HPSEC) of feces supernatant showed that the food‐grade κ‐carrageenan was not absorbed or significantly degraded in the digestive tract of obese mice. Hence, the fact that food‐grade κ‐carrageenan was not significantly metabolized by the organism and did not cause obvious dysregulation of colonic inflammatory genes provided evidences for its noncolonic toxicity in obese mice. An anti‐obesity potential of food‐grade κ‐carrageenan was probably mediated by the regulation of lipids metabolism‐related genes.

## Graphical abstract

In this study, we aimed to explore the mechanisms of carrageenan actions, and the obese mice with no treatment (HFD) or 5% food‐grade κ‐carrageenan intervention (H5%) were used to perform carrageenan metabolism experiments and colonic transcriptome sequencing. The toluidine blue (TB) staining of colon and high‐performance size exclusion chromatography (HPSEC) of feces supernatant showed that the food‐grade κ‐carrageenan was not absorbed or significantly degraded in the digestive tract of obese mice. The transcriptome sequencing showed that no genes involved in the inflammatory pathways or tight junction protein encoding were significantly dysregulated by 5% carrageenan. However, the expression of lipid metabolism genes meaningfully changed as evident by the decreased gene levels of adipocytokines, lipogenesis, along with absorption and transport, but increased adipolysis and oxidation.

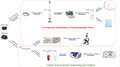

## 1 INTRODUCTION

Carrageenan, extracted from red algae such as *Chondrks crispus*, *Gigartina stdata*, and *Eucheuma* species, is a kind of plant colloid consisting of native polysaccharides (Rees, 1963; Tobacman, 2001). Carrageenan is composed of sulfated galactose and 3, 6‐dehydrated galactose, which form a carbon backbone and are linked by alternating α‐1,3 and β‐1,4 linkages. In general, carrageenan can be mainly divided into κ, ι and λ subtypes due to the different extracted sources, the galactose linkages conformation and the degree and position of sulfation (Campo et al., 2009; McKim et al., 2019; Tobacman, 2001). Carrageenan usually has a high molecular weight. The molecular weight of native carrageenan is up to 1,500 ~ 20,000 kDa, and the value for food‐grade carrageenan is also at least 100 kDa (Cohen ＆ Ito, 2002; Tobacman, 2001). Carrageenan with high molecular weight possesses excellent thickening, gelling property and protein reactivity, and is hence used widely in jelly, low‐fat meat products, soft candies, toothpaste, cleaning products, sustained‐release capsules, and cod liver oil. (He et al., 2017; Reinagel, 2015; Shang et al., 2017). Moreover, carrageenan is reported to have the physiological activities of anticoagulant, antilipidemic, antiviral, and immune regulation (Anderson et al., 1965; Campo et al., 2009; Carlucci et al., 2004; Chin et al., 2019; Shu et al., 2017). In the pharmacology and medical researches, carrageenan‐induced inflammation models are utilized to explore the pathogenesis mechanisms of inflammatory response or the effectiveness of anti‐inflammatory drugs by injecting carrageenan solution into the specific location of animals (Basu et al., 2017; Sadeghi et al., 2011; Solanki et al., 2014).

In the past decades, the edible safety of carrageenan has been highly disputed when it is used as food and medicine additive or vehicle, and the content of controversy is majorly focused on the colitis‐induction possibility of carrageenan. At present, the research conclusions and viewpoints on the intestinal toxicity of carrageenan at home and abroad are mainly divided into three kinds: (a) Carrageenan has a risk of inducing colonic inflammation or tumor (Bhattacharyya et al., 2007; Borthaku et al., 2007); (b) Carrageenan is a conditional inflammatory agent and can aggravate the intestinal toxic reaction in body with an inflammatory state, but be safe in healthy body (Bhattacharyya et al., 2017; Wu et al., 2017); (c) Carrageenan is not toxic to the intestine of host (Weine et al., 2007). The existence of such a dispute resulted from the numerous factors affecting the safety of carrageenan, such as the quality and dosage of carrageenan, and the body state of animal. The different materials and conditions adopted in different researches can draw completely different conclusions (Blakemore & Harpell, 2009; McKim, 2014). In our previous works, a series of experiments have been carried out to explore the effects of various factors on the pro‐inflammatory properties of carrageenan. The results demonstrated that up to 5% food‐grade κ‐carrageenan added to the diet did not induce colitis in both normal and obese mice. Moreover, it was observed that 5% food‐grade κ‐carrageenan or native κ‐carrageenan significantly slowed down body fat accumulation in obese mice (Chin et al., 2019; Zhang et al., 2021; Zhang et al., 2021).

The results of our previous studies mentioned above have been confirmed by other studies. For example, Weiner suggested that 2.5% and 5% κ‐carrageenan (with an average Mw of 196,000–257,000) treatment for 90 days could not cause any adverse intestinal effects in rats (Weine et al., 2007). Panlasigui and his co‐workers indicated that carrageenan could reduce blood cholesterol and lipid levels of human subjects by binding binds bile acids and reducing lipid absorption in intestine, when it was incorporated into the food items as a major source of dietary fiber (Panlasigui et al., 2003). However, these studies have not yet achieved a comprehensive and thorough investigation of the mechanism of carrageenan action through some method. Transcriptome is a high‐throughput sequencing technology that can acquire a rapid access to almost all of the transcripts and their expression level in a sample, so that wealth and complex biological information can be obtained in a single pass (Wilhelm et al., 2010). The application of transcriptomics to explore the mechanisms behind the colitis‐occurrence possibility and lipid‐metabolism regulation induced by carrageenan is scarce. Therefore, using colonic transcriptome, we aimed to explore the deeper mechanisms of food‐grade κ‐carrageenan activities in the paper.

The dietary components entering into gastrointestinal tract can be metabolized by the organism itself or microorganism of gut, while the process will affect the body itself in turn. This interaction between the organism and the substance leads to the alleged physiological activity or toxicity of one component. The metabolism of carrageenan in mice hence needs to be studied to find the reason for its actions. Here, the Toluidine Blue (TB) staining and high‐performance size exclusion chromatography (HPSEC) were used to trace the metabolic footprint of carrageenan in digestive tract, which was expected to support our conclusion combined with the transcriptome data.

Our previous results showed that 0.05 ~ 5% food‐grade κ‐carrageenan in diet were not colonic toxic in obese mice, but the highest intervention dosage of 5% had the most significant weight loss effect (Zhang et al., 2021). Therefore, in this study, the 5% carrageenan‐treated mice were chosen to be the representative to perform the colonic transcriptome sequencing and carrageenan metabolism experiment to investigate the mechanisms of noncolonic toxicity and lipid reduction effect of food‐grade κ‐carrageenan (untreated obese mice as blank control). Our results were expected to provide a novel idea for carrageenan safety research.

## 2 MATERIALS AND METHODS

### 2.1 Materials and reagents

TB dyes were provided by Servicebio Co., Ltd. TLC Silica gel 60 F254 was purchased from Merck KGaA. Trizol reagent was purchased from Thermo Fisher Scientific Inc. Transcriptome sequencing was supported by majorbio Co., Ltd.

### 2.2 Animal experiment design

Animal experimental procedure has been described in detail previously. Briefly, male specific pathogen‐free (SPF) C57BL/6Cnc obese mice were sacrificed after 6‐weeks’ intervention of 5% food‐grade κ‐carrageenan (H5%), obese mice without any treatment as blank control (HFD). Before being taken to death, all mice underwent 12‐hr fasting (Zhang et al., 2021). A total of eight mice (four for each group) were randomly selected to perform carrageenan metabolism determination and colonic transcriptome sequencing to explore the action mechanism of 5% food‐grade κ‐carrageenan, including its effect of nontoxicity to colon and inhibition to obesity.

### 2.3 Colon tissues collection and preparation

#### 2.3.1 Colon preparation for transcriptome

Approximately 1 cm of the proximal colons were harvested and conserved in liquid nitrogen, with all mice having required colons taken in a comparable site. The colon tissues were then stored in the −80℃ refrigerator for subsequent RNA extraction and transcriptome sequencing.

#### 2.3.2 Toluidine Blue (TB) staining of colon

Another colon section of each mouse (about 1 cm) was fixed in 4% paraformaldehyde instantly after harvesting. The fixed tissues were paraffin‐embedded and longitudinally cut into 4 μm slices. The TB staining was then performed. Residue of carrageenan in colonic lumen and potential absorption by epithelial cells (purple‐red) were observed under electric microscope (NIKON/Ni‐E), and 100x images were collected.

### 2.4 Colonic transcriptome assay

#### 2.4.1 RNA extraction

Total RNA of colon tissues was extracted by Trizol protocol (Mi et al., 2020). In brief, 1 cm of frozen colon segment weighting about 200 ~ 300 mg was put into 1 ml Trizol reagent and broken with beads. After 5 min incubation at room temperature, 0.2 ml chloroform was added into the homogenate and mixed. The miscible liquids were then centrifuged at a condition of 4℃ and 12,000 rpm/min after another 10 min room temperature‐incubation. The supernatant was removed and transferred into a new tube, and an equivalent volume of isopropanol was then added to precipitate nucleic acid. Extracted RNA after washing can be dissolved in DEPC water. The concentration and purity of the RNA were detected by Nanodrop 2000 (Thermo Fisher), and the integrity was detected by agar‐gel electrophoresis. The qualified RNA was diluted and for standby application.

#### 2.4.2 Transcriptome sequencing

All transcriptome sequencing and part of bioinformatics analysis were supported by majorbio Co., Ltd. Briefly, total mRNA after Oligo dT enrichment and fragmentation screening was transcribed reversely into cDNA. The cDNA can be sequenced by the Illumina Novaseq 6,000 (USA) after adaptor connection. Clean Reads of each sample were then sequence aligned with the specified reference genome (source: http://asia.ensembl.org/Mus_musculus/Info/Index) and performed gene expression analysis.

#### 2.4.3 Transcriptome bioinformatics analysis

Gene expression difference analysis: Based on the quantitative expression (Transcripts Per Million reads (TPM) as quantitative index), gene expression difference between the groups was carried out. The difference analysis software was DESeq2, and the screening threshold was |log2FC|≥1 (FC ≥ 2 or FC ≤ 0.5) & *p* value < 0.05.

Enrichment analysis: R scripts or Goatool software was used to carry out KEGG PATHWAY or GO TERM enrichment analysis on the differential genes, so as to obtain the major KEGG pathways or GO functions of these genes. Fisher's exact test was applied to show that there was significant gene enrichment on certain KEGG PATHWAY or GO TERM when *p*‐adjust < 0.5.

Correlation analysis between genes and phenotypes: Weighted Gene Co‐Expression Network Analysis (WGCNA) function was used to analyze the correlation between the significant different phenotypes (body weight and body fat rate, Table S1) and all of the differential genes between the two groups. Briefly, the genes/transcripts after background correction and standardization can be classified and form modules according to their expression patterns. Finally, the constructed modules were identified and the key modules were obtained through association analysis with phenotypic data. Correlation coefficient (Corr) was analyzed by Spearman method and Corr ≥ 0.8 & *p* < 0.05 was considered as a significant correlation.

### 2.5 Feces collection and carrageenan degradation measurement

#### 2.5.1 Thin Layer Chromatography (TLC)

The day before sacrifice, feces of mice were collected in sterile tubes and stored in −80℃ immediately. After freeze‐drying, the mixed feces of mice in each group were ground into powder in a mortar, and 50 ml/g of ultra‐pure water was then added to prepare into a suspension. The pH was adjusted to 8 by 1 M NaOH for protein precipitation and removal. After that, the suspension was heated in a hot water bath at 70℃ for 15 min, and then centrifuged at 6,000 rmp for 15 min to take the supernatant for use (Arakawa et al., 1988; Li, 2014; Pittman et al., 1976; Tache et al., 2000). Carrageenan solution was also prepared for TLC. The high‐efficiency chromatography silica gel plate was cut into an appropriate size and 0.2 μl feces supernatant or carrageenan solution were sampled on it. The plates were then placed in the unfolding agent (formic acid: N‐butanol: water = 6:4:1) and then blow‐dried. The plates were soaked in the orcinol agent and blow‐dried. The silica gel plates were finally heated in a 120℃ drying oven for 3 min for color development (Li, 2014).

#### 2.5.2 High performance size exclusion chromatography (HPSEC)

The molecular weight and distribution of carrageenan sample and carrageenan in feces were further detected by HPSEC. The protocol was listed as following: the carrageenan sample was diluted to 0.5 mg/ml by mobile phase (0.2 M sodium chloride solution with 0.003 M sodium azide) and filtered through a 0.2 μm membrane. The prepared sample of 0.8 ml was injected into the gel chromatography instrument equipped with multiangle laser scatterometer and differential refraction detector to be measured, with the velocity of mobile phase and column temperature of 0.5 ml/min and 30℃, respectively (Arakawa et al., 1988; Uno et al., 2001). A 0.4 ml of prepared fecal supernatant above was taken and mixed with the mobile phase (vol/vol 1:1) to determine the degradation of carrageenan in feces of H5% mice (Tache et al., 2000).

### 2.6 Statistical analysis

The amount of gene expression (TPM) was expressed as Mean ± *SEM* (standard error of mean). The expression difference was analyzed by DESeq2 and the screening threshold of statistical significance was |log2FC|≥1 (FC ≥ 2 or FC ≤ 0.5) & *p* value < 0.05; The enrichment analysis was performed by Fisher’ exact test and *p*‐adjust < 0.5 was considered as significant enrichment; The correlation coefficient between phenotypes and genes were analyzed by Spearman and Corr ≥ 0.8 & *p* < .05 was considered as significant correlation.

## 3 RESULTS

### 3.1 The metabolism of 5% food‐grade κ‐carrageenan in gastrointestinal tract of obese mice

**FIGURE 1:**
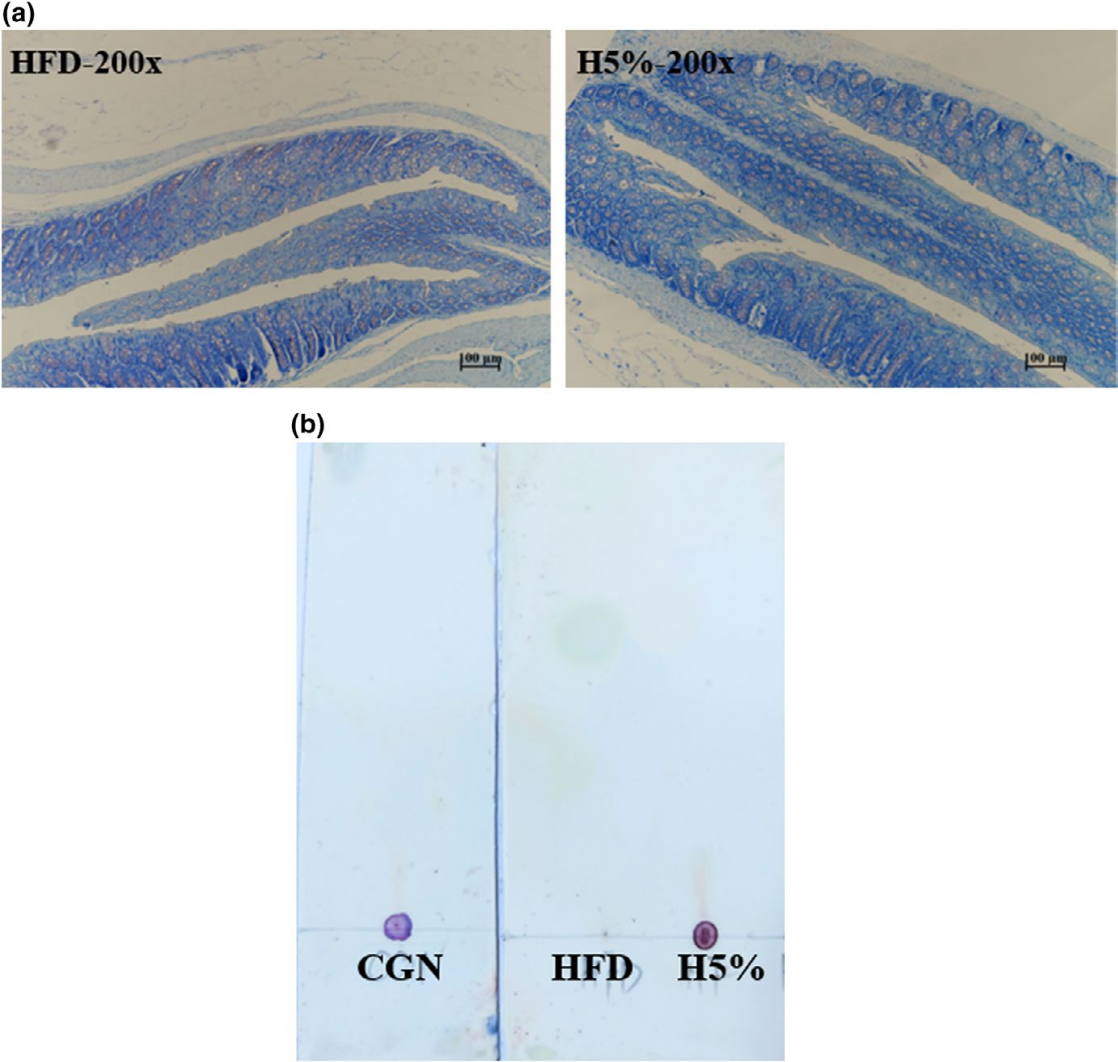
The Residual and Degradation of 5% Food‐grade κ‐Carrageenan in Colon and Feces. (a) TB staining of colon (200x). (b) TLC of carrageenan sample and fecal supernatant of mice

TB staining was performed to detect carrageenan residual in colon lumen as well as potential phagocytosis by epithelial cells. As Figure 1a shown, there was no visible residue of purple‐red material in the colonic lumen of H5% mice, which suggested that carrageenan excreted with feces after 12 hr of fasting and little remained in the colon. Furthermore, as can be seen from the staining figures, there was little difference in cellular and glandular staining between the two groups. The goblet cells and mast cells of colon in both groups were purple‐red because of the existence of heparin, histamine, or other sulfate polysaccharides mucus. However, no heterochromic purplish‐red had been observed in the epithelial cells of colonic mucosa of treated mice, suggesting that carrageenan was not phagocytosed or absorbed by the colon (Agarwal et al., 2010; Weiner, 2014).

The degradation condition of carrageenan in feces of mice was analyzed by TLC and HPSEC (Figure 1b and Table 1). The results showed that neither the carrageenan solution (CGN) nor the fecal supernatant of H5% mice (H5%) unfolded on the chromatography plate, rough indicating that low‐molecular fractions were almost absent in the feces. While the fecal supernatant of mice in blank group (HF

D) showed no color on the baseline due to the absence of carrageenan. HPSEC further demonstrated that carra

geenan scarcely degraded when passing the digestive tract of mice. Compared to the original carrageenan sample, the carrageenan in feces of H5% mice had a similar average molecular weight (300.4 kDa versus 287.2 kDa), with 200 ~ 400 kDa macromolecule still owing predominant percentage. As Table 1, it could also be elicited that a small amount of 400 ~ 800 kDa carrageenan in feces of H5% mice is degraded into relatively smaller ones, including 4.7% of 200 ~ 400 kDa, 0.6% of 100 ~ 200 kDa and 0.4% of 50 ~ 100 kDa molecules, but no <50 kDa degraded‐carrageenan appeared.

**TABLE 1:** The Molecular Weight and Distribution of Carrageenan in Feces of Mice by HPSEC

Group Molecular weight and distribution	CGN	H5%
<50 kDa	0.0%	0.0%
50 ~ 100 kDa	0.0%	0.4%
100 ~ 200 kDa	0.0%	0.6%
200 ~ 400 kDa	86.0%	90.7%
400 ~ 800 kDa	13.3%	7.1%
>800 kDa	0.7%	1.2%
Average Mw	300.4 kDa	287.2 kDa

Our results demonstrated that food‐grade κ‐carrageenan stayed in the intestine for only a few hours, after which it directly excreted with the feces and remained essentially unchanged. Meanwhile, during the residence time in intestine, carrageenan was not sufficiently phagocytized or degraded. These findings provided evidences and supports for the conclusion that food‐grade κ‐carrageenan was not toxic to the colon of obese mice (McKim et al., 2019). Besides, the production of a small amount of 50 ~ 200 kDa carrageenan could serve as metabolic substrates for intestinal flora, which may be one of causes of body weight and fat loss in obese mice.

### 3.2 Different expression of the colonic gene after 5% food‐grade κ‐carrageenan treatment

**FIGURE 2:**
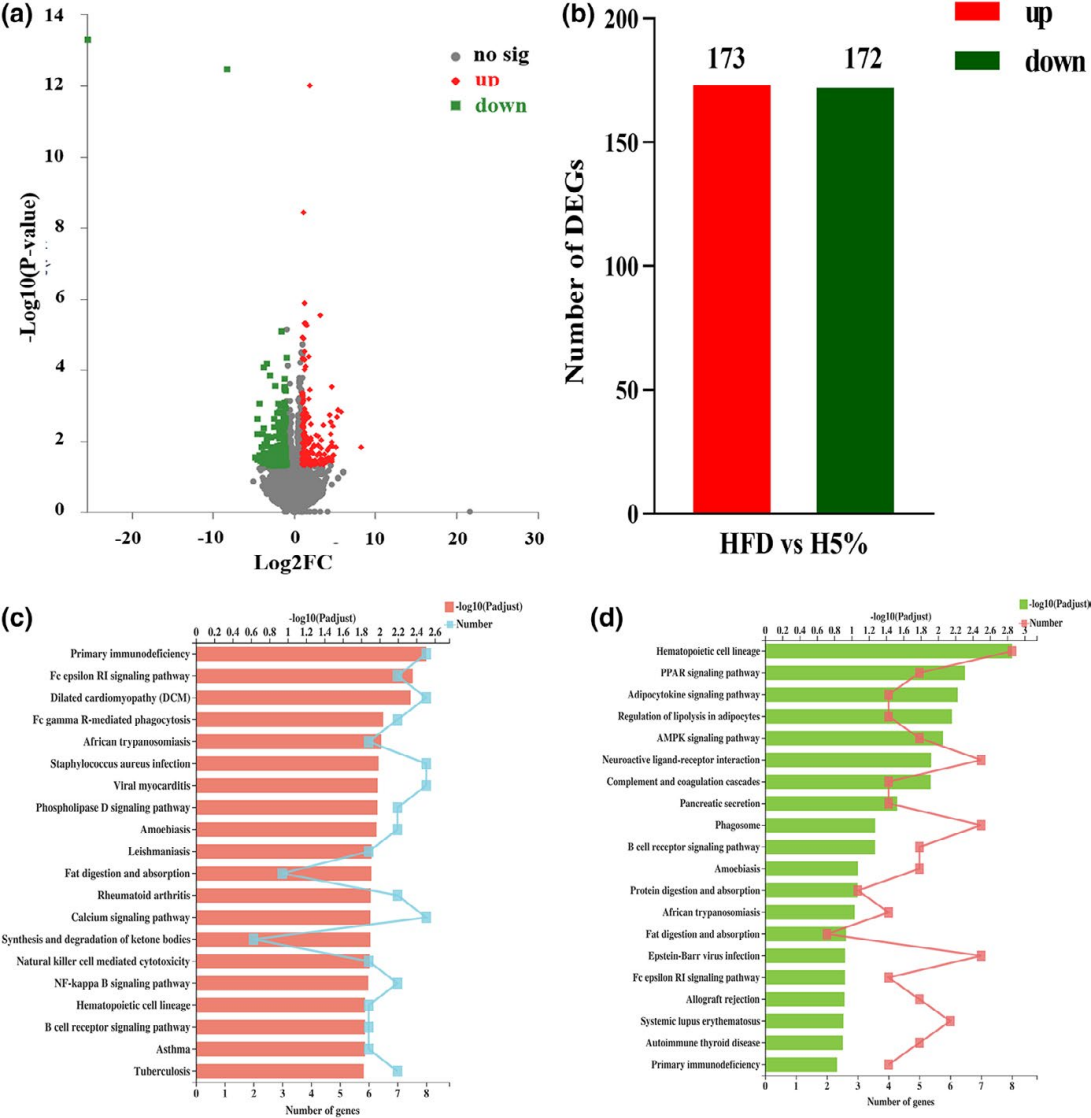
5% Food‐Grade κ‐Carrageenan‐induced Colonic Denes Dysregulation of Obese Mice. (a) Volcanic map of gene expression between groups. (b) The numbers of up‐regulated and down‐regulated genes. (c) Up‐regulated gene enrichment by KEGG PATHWAY. (d) Down‐regulated gene enrichment by KEGG PATHWAY. TMP as expression index, *n* = 4. DESeq2 software was used to analyze the gene difference between groups, |log2FC|≥1 & *p* < 0.05; The Fisher's exact test was used to perform enrichment analysis, *p*‐adjust < 0.5, the top 20 was shown

The results of transcriptome sequencing showed that colonic genes of mice were differently expressed after 5% food‐grade κ‐carrageenan treatment compared to the blank group. As Figure 2a, the rigorous screening criteria of *p* < 0.05 and |log2FC|≥1 were adopted and the genes meeting this were considered to be differentially expressed compared with the blank group. The red dots represented up‐regulated genes and green for down‐regulated ones, with dots near upper left and right having the most significant expression difference (a larger |log2FC| and ‐log10 (*p*‐value)). The detailed information of differently expressed genes, together with their fold changes and GO annotations were all listed in Table S1‐S2. In brief, after 5% food‐grade κ‐carrageenan intervention, a total of 345 colonic genes were dysregulated and 173/172 were respectively up‐regulated and down‐regulated (Figure 2b). Functional annotation of differential genes by KEGG PATHWAY indicated that 38 signaling pathways were altered in H5% mice, including the pathways of metabolism, genetic information processing, environmental information processing, cellular processes, organismal system, and human diseases, but no obvious inflammatory pathways were observed (Data not shown).

To obtain the primary pathways that differential genes got involved in, the up/down‐regulated genes were respectively enriched by KEGG analysis and the top 20 signaling pathways were displayed. As Figure 2c‐d shown, plentiful enriched pathways were concentrated in the glucose, lipid and energy metabolism. As Figure 2c, the up‐regulated genes could be enriched in the pathways of lipid metabolism (Phospholipase D signaling pathway, ‐log10(*p*‐adjust) = 1.98), lipid digestion and absorption (‐log10(*p*‐adjust) = 1.91) and glucose metabolism (NF‐κB signaling pathway, ‐log10(*p*‐adjust) = 1.88). Moreover, it was observed that down‐regulated genes were more enriched in lipid metabolism functions, such as fatty acid oxidation (PPAR/AMPK signaling pathway, ‐log10(*p*‐adjust) = 2.06/2.31), adipocytokine release (Cytokines signaling pathway, ‐log10(*p*‐adjust) = 2.23), lipid catabolism (Regulation of lipolysis in adipocytes, ‐log10(*p*‐adjust) = 2.16), and lipid digestion (Lipid digestion and absorption/Pancreatic secretion, ‐log10(*p*‐adjust) = 0.93/1.53), all of which had strong significance (high bar chart) (Figure 2d). These evidences indicated that 5% food‐grade κ‐carrageenan had an intense impact on the glucose and lipid metabolism in obese mice, which was consistent with the results of our previous experiments (Zhang et al., 2021).

In addition, the figures also showed that some immune‐related pathways were enriched, such as Primary immunodeficiency and B cell receptor signaling pathway. The obvious immune‐regulatory effect of carrageenan has been proved by many studies (MallettIan et al., 1985; Thomson & Fowler, 1981). In conclusion, 5% food‐grade κ‐carrageenan can lead to differential expression of colonic genes in obese mice, and these genes are mainly involved in lipid metabolism and immune regulation, which may provide ideas for us to find the mechanism of obesity inhibitory effect of food‐grade κ‐ carrageenan.

### 3.3 The inflammatory genes changes in colon after 5% food‐grade κ‐carrageenan treatment

The above annotation analysis of all sequenced genes had shown that no obvious inflammatory signaling pathways were activated by 5% carrageenan. We still further analyzed the expression differences of colitis‐related genes between HFD and H5%, including pathway genes by which carrageenan may trigger colitis, inflammatory factors, pro‐inflammatory chemokines and genes expressing tight junction protein.

As shown in Table 2, the results of colonic transcriptome demonstrated that the expression of genes involved in inflammatory pathways (TLR4‐Bcl10 and ROS), such as Toll‐like receptor 4 (TLR4), B‐cell lymphoma/leukemia‐10 (Bcl‐10), inhibitor of κB kinases (IKKγ and IKKβ), inhibitor α of κB (IκBα), and nuclear transcription factor‐κB (NF‐κB) were not observed to be significantly differential between HFD and H5% mice (*p* > 0.05 or |log2FC|<1). The genes expression of inflammatory factors in the downstream pathway, including tumor necrosis factor‐α (TNF‐α), interleukin‐6 (IL‐6), interleukin‐1β (IL‐1β), interleukin‐2 (IL‐2), interferon‐γ (INF‐γ), interleukin‐4 (IL‐4), interleukin‐10 (IL‐10), Th17 cytokine (Th17A), and pro‐inflammatory chemokines (Ccl and Cxcl family) were all similar between blank group and 5% carrageenan‐treated group (*p* > 0.05 or |log2FC|<1, Table 3, 4). In addition, the expression of genes encoding tight junction protein in intestinal epithelial cell was also counted. The data in Table 5 depicted that compared to the blank control, the genes expression of zonula occluden‐1 (ZO‐1), occludin (Ocln), claudins (Cldn1 and Cldn4), and junctional adhesion molecules (JAM‐2 and JAM‐3) were only lightly up‐regulated (1<FC < 2) or downregulated (0.5<FC < 1) by 5% carrageenan, but did not reach the predetermined threshold for significant difference (|log2FC|≥1 & *p* < 0.05). In short, the results of colonic transcriptome indicated that 5% food‐grade κ‐carrageenan added into diet could not induce an intense dysregulation of inflammatory genes in colon, which was another reason why 5% food‐grade κ‐carrageenan had no risk of colitis in obese mice.

**TABLE 2:** The Expression of Genes Involved in the Inflammatory Pathway by Transcriptome Sequencing

Gene Group/index	TLR4	Bcl−10	IKKγ	IKKβ	IκBα	NF‐κB
HFD (TPM)	5.6550 ± 0.5125	26.3700 ± 1.3966	7.6125 ± 0.4876	16.4175 ± 1.0971	46.7325 ± 2.7261	28.3950 ± 2.6119
H5% (TPM)	3.6900 ± 0.5475	24.1725 ± 1.2308	5.8900 ± 1.2480	12.8275 ± 0.9771	45.4875 ± 2.6700	22.6900 ± 1.5265
FC (H5%/HFD)	0.727	1.061	0.86	0.868	1.145	0.909
*p* value	0.0216	0.6039	0.2357	0.1545	0.2090	0.3545

Note

Gene expression represented as mean ± *SEM*, TPM as expression index, *n* = 4, *p* > .05 or |log2FC|<1.

**TABLE 3:** The Genes Expression of Inflammatory Factors in the Downstream Pathway by Transcriptome Sequencing

Gene Group/index	TNF‐α	IL−6	IL−1β	IL−2	INF‐γ	IL−4	IL−10	IL−17A
HFD (TPM)	0.7500 ± 0.2347	0.0850 ± 0.0293	0.5775 ± 0.1045	0.0700 ± 0.0226	0.2150 ± 0.0611	0.0850 ± 0.0736	0.0700 ± 0.0297	0.0000 ± 0.0000
H5% (TPM)	0.7225 ± 0.2730	0.0450 ± 0.0266	0.5000 ± 0.1155	0.0375 ± 0.0225	0.3175 ± 0.1207	0.1275 ± 0.0756	0.0925 ± 0.0114	0.0125 ± 0.0108
FC (H5%/HFD)	1.276	0.552	0.974	0.676	0.717	1.899	1.545	1.724
*p* value	0.6409	0.6286	0.9438	0.7560	0.6033	0.7206	0.6070	0.8240

Note

Gene expression represented as mean ± *SEM*, TPM as expression index, *n* = 4, *p* >.05 or |log2FC|<1.

**TABLE 4:** The Gene Expression of Pro‐inflammatory Chemokines by Transcriptome Sequencing

Gene Group/index	Ccl2	Ccl3	Ccl4	Ccl5	Cxcl2	Cxcl10
HFD (TPM)	1.1625 ± 0.2629	0.6500 ± 0.1344	1.2100 ± 0.4125	65.3925 ± 8.5078	0.1800 ± 0.0840	3.8400 ± 0.6388
H5% (TPM)	1.1025 ± 0.2988	0.6750 ± 0.2993	1.6925 ± 0.4116	77.7450 ± 17.4486	0.1200 ± 0.0813	2.6050 ± 0.4507
FC (H5%/HFD)	1.015	1.385	1.730	1.451	0.254	0.754
*p* value	0.9698	0.5864	0.2602	0.2172	0.8020	0.2421

Note

Gene expression represented as mean ± *SEM*, TPM as expression index, *n* = 4, *p* >.05 or |log2FC|<1.

**TABLE 5:** The Expression of Genes Encoding Tight Junction Protein by Transcriptome Sequencing

Gene Group/index	ZO−1	Ocln	Cldn1	Cldn4	JAM−2	JAM−3
HFD (TPM)	17.1050 ± 0.9507	37.6200 ± 2.3637	0.9250 ± 0.1495	33.7875 ± 5.0359	8.4675 ± 0.1621	10.5750 ± 0.5563
H5% (TPM)	11.4500 ± 2.1867	27.1800 ± 3.6015	0.5125 ± 0.1018	30.9575 ± 6.1752	8.2850 ± 1.1924	11.3900 ± 1.8618
FC (H5%/HFD)	0.675	0.8150	0.611	1.154	1.048	1.180
*p* value	0.0103	0.0868	0.0519	0.6091	0.7353627	0.2693

Note

Gene expression represented as mean ± *SEM*, TPM as expression index, *n* = 4, *p* > .05 or |log2FC|<1.

### 3.4 Correlation analysis of physiological phenotypes and differential genes

**FIGURE 3:**
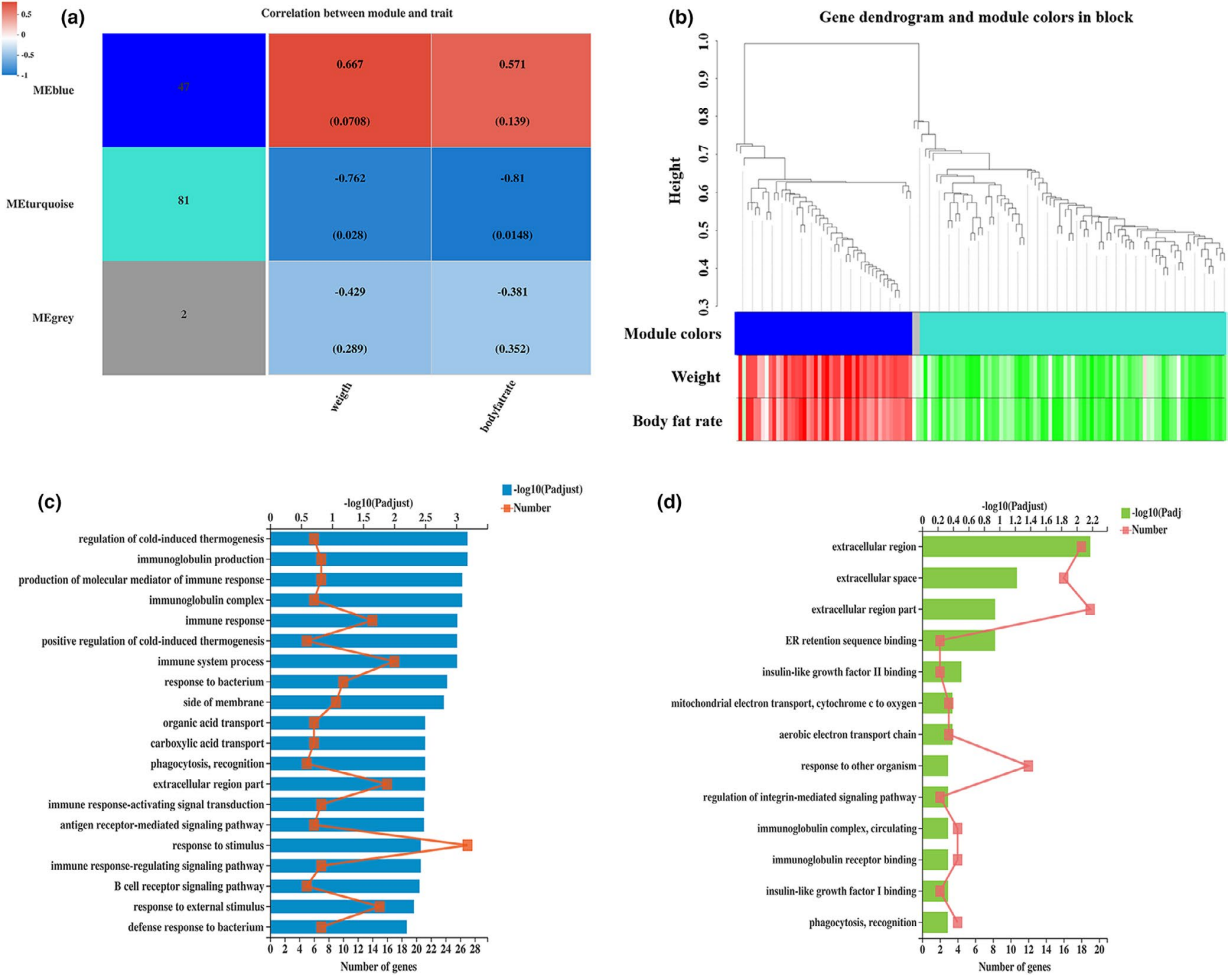
Correlation Analysis of Physiological Phenotype and Differential Genes. (a) The correlation analysis between modules and phenotypes. (b) The cluster analysis between genes and phenotypes. (c) The GO enrichment analysis of positively correlated genes. (d) The GO enrichment analysis of negatively correlated genes. *n* = 4. The method of Spearman was used to calculate correlation coefficient, corr ≥ 0.8 & *p* < 0.05 regarded as significant correlation; The Fisher test was used to perform enrichment analysis, *p*‐adjust < 0.5, the top 20 was shown

As shown in Figure 3a and Table 6, based on the expression pattern and the correlation with phenotypes (The body weight and fat rate data of mice presented in Table S2), all differential genes (after data preprocessing, 130) were mainly classified into two characteristic modules by WGCNA, blue and turquoise modules. There were 47 gene members in blue module, which showed a positive correlation with the phenotypes (shown in red). The Correlation between the blue module and the body weight or body fat rate was 0.667 and 0.571, and the *p*‐values were 0.078 and 0.139 respectively, which exhibited a passable but not significant correlation (Corr < 0.8 or *p* > 0.05). The turquoises module was negatively correlated with the phenotypes, which was represented by dark blue. The correlation coefficients with the phenotypes were −0.762 and −0.81 respectively, and the *p*‐values were 0.028 and 0.0148, showing a significant correlation (Corr ≥ 0.8 & *p* < 0.05). Figure 3b displayed the cluster analysis between genes and phenotypes, which further showed the correlation between genes in modules and phenotypes. The upper part of the figure was the hierarchical cluster tree of genes, the middle part were the modules consisting of genes, and the lower part showed the heat map of correlation between genes and phenotypes. In the heat map, red represented positive correlation and green represented negative correlation, so it could be seen that almost all gene members in each module were well correlated with the phenotypes. In other words, genes in the both modules participated in the process of fat reduction and weight loss.

**TABLE 6:** The Statistical Table of Correlation Between Modules and Phenotypes

Module	Gene number	Corr (W/FR)	*p*‐value (W/FR)
blue	47	0.667/0.571	0.0708/0.139
turquoise	81	−0.762/−0.81	0.028/0.0148

As Figure 3c, GO enrichment analysis was performed separately for the above positively and negatively correlated gene sets to obtain the main molecular functions played by these genes. The results showed that the genes positively correlated with the phenotypes were mainly concentrated in the immune function of the body, such as immune response, B cell receptor signaling pathway, immunoglobulin production and immune response‐regulating signal transduction, suggesting that 5% food‐grade κ‐carrageenan had an effect on the immune system of obese mice. The change of body immunity is often accompanied by the change of body fat, such as an increased IgA level after weight loss (Yang and Park, 2010; Zamarron et al., 2015). Moreover, it's observed that, some genes significantly negatively correlated with the phenotypes were focused on the insulin‐like growth factor Ι and Ⅱ (IGFs) binding function, indicating that 5% food‐grade κ‐carrageenan could restore the obesity‐induced IGFs decline and eased the symptom (Deodati et al., 2013; Yaylali et al., 2010).

In conclusion, after 5% food‐grade κ‐carrageenan intervention, the phenotypic changes of mice showed a good correlation with differential genes, especially a significant negative correlation with IFGs expression level and functional exertion, suggesting that 5% food‐grade κ‐carrageenan played the role of anti‐obesity by possibly affecting the level of regulatory factor or immune response.

### 3.5 A dysregulation of lipid metabolism‐related genes in colon after 5% food‐grade κ‐carrageenan intervention

**FIGURE 4:**
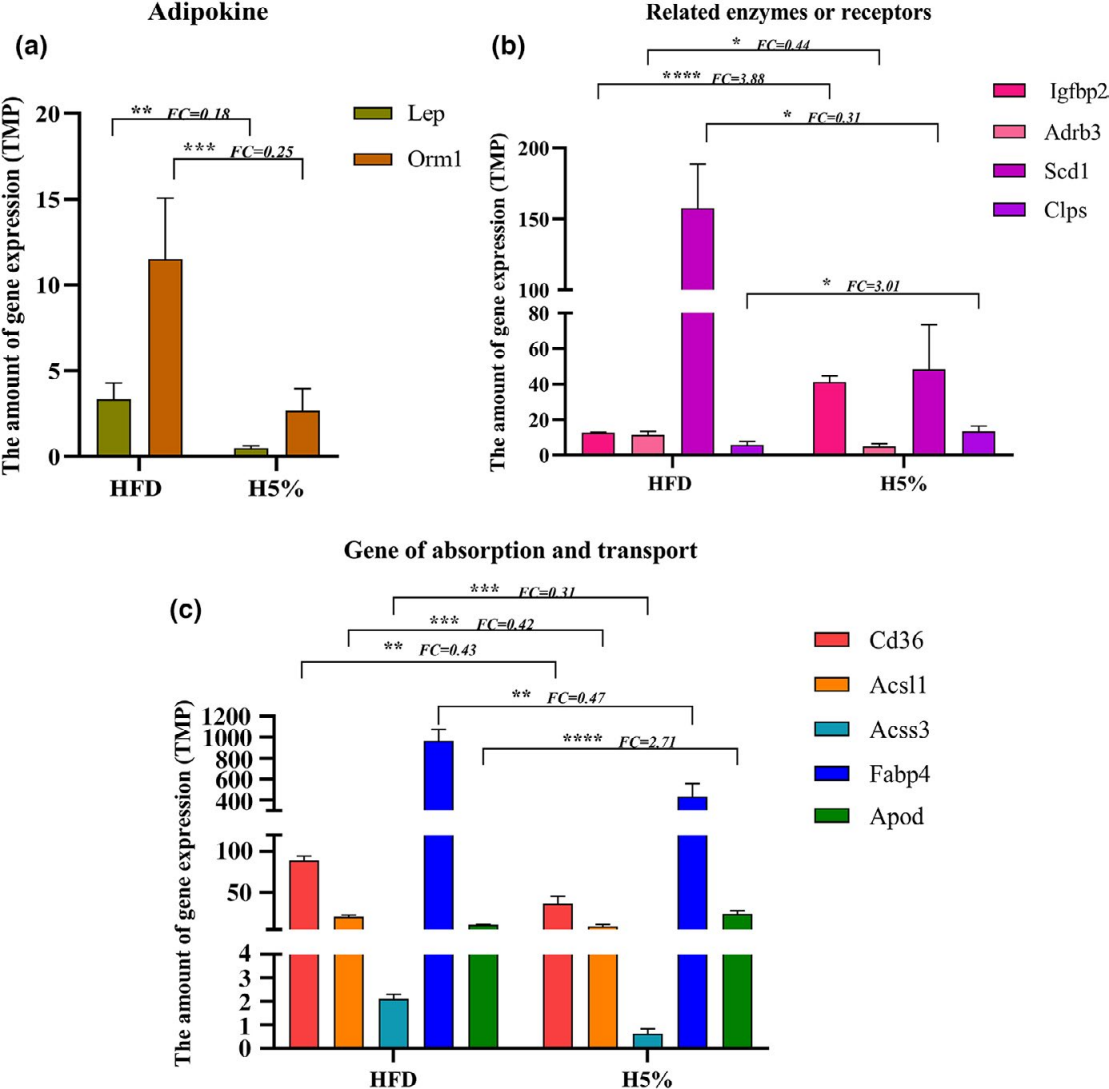
The effect of 5% food‐grade κ‐carrageenan on the expression of lipid metabolism‐related genes in colon. (a) The effect on the gene expression of adipokines. (b) The effect on the gene expression of lipid metabolism‐related enzyme and receptor. (c)The effect on the gene expression of lipid absorption and transport. TMP as expression index, *n* = 4. DESeq2 software was used to analyze the differences among groups, |log2FC|≥1 & ^*^ *p* < 0.05, ^**^ *p* < 0.01, ^***^ *p* < 0.001

To further investigate the mechanism of reduced body fat accumulation by 5% food‐grade κ‐carrageenan in obese mice, lipid metabolism‐related genes that were significantly differentially expressed between the H5% and blank groups were selected and analyzed separately. As Figure 4a depicted, the gene expression levels of leptin (Lep, FC = 0.18) and orosomucoid‐1 (Orm1, FC = 0.25) in colon of mice of H5% group were significantly lower than those of blank group (*p* < 0.01, *p* < 0.001 & FC < 0.5), suggesting that 5% food‐grade κ‐carrageenan reduced the gene expression and possible protein excretion of adipocytokines, and thus slowed down the accumulation of body fat caused by high‐fat diet. In addition, it was found that the expression of stearoyl‐CoA desaturase‐1 (Scd1) gene was significantly decreased (*p* < 0.05 & FC = 0.31) and colipase (Clps) increased (*p* < 0.05 & FC = 3.01) in the H5% group, indicating that 5% food‐grade κ‐carrageenan inhibited obesity through regulating fat synthesis and hydrolysis (Figure 4b). Interestingly, the significantly increased expression of insulin like growth factor binding protein 2 (Igfbp2) (*p* < 0.00001 & FC = 3.88) after 5% carrageenan treatment was highly consistent with the above‐mentioned results that some genes negatively associated with the phenotypes were involved in IGF binding function. The gene expression of Adrenergic receptor‐β3 (Adrb3) in the colon of H5% mice had a 0.44‐fold reduction compared to the HFD mice (*p* < 0.05), which also corresponded to the reduced serum lipid levels in our animal experiment (Figure 4b) (Zhang et al., 2021).

As shown in Figure C, the colonic transcriptome also showed that the expression of the genes related to lipid transport and absorption were significantly reduced in the H5% group. Lots of studies have shown that the reduced expression of these genes is associated with decreased lipid utilization and increased lipid consumption. It is observed in our results that fatty acid translocase (FAT, Cd36) gene expression in colon of H5% mice was significantly decreased to an extent of 0.43‐fold that of HFD mice (*p* < 0.01). The transcriptome also demonstrated a significant decrease of fat acid binding protein 4 (Fabp4) gene level after 5% carrageenan intervention (FC = 0.47, *p* < 0.01). Therefore, the expression decline of the Cd36 and Fabp4 genes suggested that 5% food‐grade κ‐carrageenan inhibited lipids entrance and subsequent synthesis in cells, thereby suppressing lipid accumulation in obese mice. Moreover, the gene expression of acyl‐CoA synthases (Acs), including long chain acyl‐CoA synthase (Acsl1) and short chain acyl‐CoA synthase (Acss3), were also found to be significantly decreased in colon of H5% mice compared to the HFD group, with the respective fold change of 0.42 and 0.31 (*p* < 0.001, Figure 4c). Acs plays key roles in the fatty acid metabolism, the decreased gene expression of which can increase β‐oxidation and decrease TG synthesis. The Apolipoprotein (Apod) is also involved in lipid metabolism and plays a role in the hydrolysis of triglycerides. Compared with HFD, the colonic Apod gene expression of H5% mice were significantly increased, and the multiple change was 2.71. Therefore, the results of our study revealed that 5% carrageenan‐induced decrease in Acs gene levels and increase in Apod might contribute to lipid consumption but impair lipid storage.

The results of our study demonstrated that 5% food‐grade κ‐carrageenan produced obvious impact on the genes expression involved in lipid metabolism in obese mice. The decreased genes expression of adipocytokines, decreased expression of genes involved in lipid transport and synthesis, but increased genes level of lipid hydrolysis and oxidation may explain the effect of carrageenan on weight and fat loss.

## 4 DISCUSSION

Our previous studies have shown that high molecular‐weight carrageenan alone has no risk to induce colitis, but can play significant roles in fat and weight loss in obese organisms (Chin et al., 2019; Zhang et al., 2021). The researchers who hold that the carrageenan has no intestinal toxicity believe that the carrageenan is neither significantly degraded in the intestinal tract nor absorbed by the organism, but is excreted directly within 24 hr (Weiner, 2014). In his review, Weiner summarized multiple methods for determining the absorption condition of carrageenan by the intestine or organism, including TB staining, Alcian blue staining, radiolabeling, and intestinal permeability measurement. The studies using these methods showed that carrageenan did not enhance intestinal permeability and did not enter the liver through the intestine (Weiner, 2014). In our study, TB staining was used to determine the carrageenan absorption in the intestine, and the results showed that carrageenan was not phagocytized or absorbed by colonic epithelial cells, but was excreted with the feces (Agarwal et al., 2010). Although it could be roughly seen by TB staining that no carrageenan remained in the intestinal lumen of mice after a 12‐hr fast, our experiment failed to determine the total carrageenan content in the feces, so it could not absolutely sure that carrageenan had been completely excreted. The related experiments will be conducted in future studies. The transcriptome showed that, the genes expressing tight junction protein were not significantly downregulated in the colon of H5% mice, demonstrating that food‐grade κ‐carrageenan did not increase intestinal permeability and cause inflammation caused by the entry of other molecules into the intestine (Bernd & Caspary, 1989). In addition, the degradation of carrageenan in intestine of mice was determined by HPSEC, the results of which showed that the average molecular weight and distribution of carrageenan in feces of H5% mice were similar to the original carrageenan samples (Arakawa et al., 1988; Uno et al., 2001). Degraded‐carrageenan or poligeenan with molecular weight <50 kDa is considered to be an important cause of intestinal toxicity. According to the stipulation of international agencies, carrageenan used as food or medicine should contain no more than 5% of <50 kDa fragments (European Commission, 2003; JECFA & WHO, 2007). It was noted that the content of carrageenan fragments with molecular weight <50 kDa in the feces of H5% mice was 0% in our study, further indicating that food‐grade κ‐carrageenan was not significantly degraded in the intestinal tract, let alone producing intestinal toxicity. Not only that, the colonic transcriptome showed that 5% food‐grade κ‐carrageenan did not induce significant abnormal regulation of genes involved in the inflammatory pathways (TLR4‐Bcl10‐NF‐κB and ROS‐NF‐κB), genes of downstream inflammatory factors (pro‐inflammatory factors, Th1, Th2, and Th17 immune factors) and proinflammatory chemokines (Ccl and Cxcl families) in the colon of obese mice. Overall, the mechanism by which food‐grade κ‐carrageenan is not enterotoxic is that it is neither absorbed nor significantly degraded by the intestine, nor does it cause significant abnormal regulation of intestinal inflammatory genes.

The colonic transcriptomic data showed that genes significantly different between the two groups were not involved in the inflammatory response, but were mainly enriched in lipid and energy metabolism pathways. Consistent with this, the differential genes in H5% mice were well correlated with the phenotype of reduced body fat. Specially, the negative correlation module reached significant correlation (corr ≥ 0.8 & *p* < 0.05), and its gene members could be focused on the IGF binding function. Additionally, to further explore the mechanism by which 5% food‐grade κ‐carrageenan mitigates body fat accumulation, we analyzed the lipid metabolism genes that had significant difference between the two groups (Figure 4). The data showed a meaningful decline of adipocytokines, a significant regulation of lipid metabolism‐related enzymes and receptors, and dramatic expression changes of lipid absorption and transport genes in colon of H5% mice. In a state of obesity, the excessive accumulation of adipose tissue will release a series of active adipocytokines and lead to chronic inflammation and metabolic disorders (Jung & Choi, 2014). The expression reduction of Lep and Orm1 in H5% mice implied the remission of obesity and metabolic syndrome (Lee et al., 2010). Furthermore, it was possible that 5% carrageenan promoted lipid hydrolysis but inhibited lipid synthesis as evident by elevated enzymes expression of Scd1 and lowered Clps (Alberto and Luciano, 2012; Macdonald et al., 2008; Miller et al., 2009). Moreover, because the IFG secretion and binding were usually inhibited with the onset of excessive fat storage, the increased expression of Igfbp2 and the regulation of IGF binding function by 5% carrageenan suggested the relieving of obesity (Deodati et al., 2013; Yaylali et al., 2010). Adrb3 has been reported to be associated with lipid metabolism disorders, and its gene expression increases with elevated LDL level and hypertriglyceridemia, but is normal in the lean body (Alberto and Luciano, 2012; Krief et al., 1993). The decreased gene expression of Adrb3 in H5% mice agreed with their lean body shape. Crucially, the genes participating in lipids absorption and transport were analyzed. Cd36 is a fatty acid translocase widely expressed on the surface of cells that binds long‐chain fatty acids, natural or oxidized lipoproteins and facilitates their entry into cells. In the proximal intestine, Cd36 mainly ingests FA and causes it to form chylomicron (Nassir et al., 2007). Fabp4 is a subtype of intracellular fat‐binding protein, and also responsible for the binding and transport of extracellular fatty acids, which can promote lipid synthesis by its increasing expression in obese body (Kucharski & Kaczor, 2017; Qiao et al., 2019; Su et al., 2015). Therefore, the results of significant expression decline of Cd36 and Fabp4 in colon of H5% mice in our study indicated that 5% food‐grade κ‐carrageenan suppressed the absorption, utilization and transport synthesis of lipids, which was consistent with Panlasigui's results to a certain extent (Panlasigui et al., 2003). As reported, Acsl1 deficiency leads to enhanced β‐oxidation and diminished TG synthesis, while Apod deficiency results in defects in TG metabolism (Kamilah et al., 2014; Yan et al., 2015). Hence, it could be the expression decline of Acs and raise of Apod that contributed to lipid consumption and slowed lipid storage in H5% mice, which is consistent with the findings of Chin. Chin et al demonstrated that extracted native κ‐carrageenan promoted β‐oxidation of lipid by increasing genes expression of Aco and Adipors in liver and colon (Chin et al., 2019). Altogether, 5% food‐grade κ‐carrageenan might decrease lipid absorption, utilization and transport synthesis, but increase oxidative consumption, which led to retarded body fat accumulation and metabolic syndrome.

Overall, the dysregulation of lipid metabolism‐related genes mentioned above revealed the mechanism by which 5% food‐grade κ‐carrageenan alleviated obesity and metabolic syndrome. Although fat digestion and absorption mainly occur in the small intestine, studies have shown that there were still 14% of the epithelial cells performing nutrient absorption functions in colon (Wang et al., 2019). Wang et al. found that the large intestine, especially the colon, is primarily responsible for the transport and absorption of small molecules, such as metal ions, nucleotides or nucleotide sugars, ketone bodies, and aldehydes (Wang et al., 2019). A partially hydrolyzed lipids can also be absorbed by colonic epithelial cells and form chylomicron, especially in the ascending colon. Henceforth, the expression changes of lipid metabolism‐related genes in colon could also explain to some extent the weight loss effect of food‐grade κ‐carrageenan. Nonetheless, it remains to be further explored whether the expression of these genes in small intestinal epithelial cells, adipocytes or liver cells is consistent or synergistic with that in the colon. On the other hand, Chin et al. showed that after the intervention of carrageenan in obese mice, the fecal flora and SCFA composition changed significantly (Chin et al., 2019). It has long been proven that short‐chain fatty acids, such as lactate and acetate, could regulate lipid metabolism (Arau´jo et al., 2020). Our molecular weight determination of fecal carrageenan indicated that although carrageenan was not significantly degraded in the gastrointestinal tract, the production of a small amount of 50 ~ 200 kDa carrageenan might be used as substrate for fecal bacteria to synthesize SCFAs. However, the changes of fecal flora and SCFAs after food‐grade κ‐carrageenan intervention, and whether these changes are related to the production of 50~200 kDa carrageenan, all need to be further verified by subsequent experiments.

In conclusion, our results suggested that 5% food‐grade κ‐carrageenan could neither be absorbed nor be significantly degraded by the intestine, nor could it cause obvious dysregulation of colonic inflammatory genes, which provided evidences for the absence of colonic toxicity of carrageenan in obese mice. The mechanism of anti‐obesity effect of food‐grade κ‐carrageenan was probably mediated by the expression change of intestinal genes participating lipid metabolism, such as the reduced lipids availability and synthesis and raised hydrolysis and consumption. Our study provided a mechanistic explanation for the carrageenan activities, including its intestinal nontoxicity and obesity inhibition, which contributed to a clearer understanding of the edible safety of carrageenan for people, and subsequently a better application of carrageenan.

## CONFLICT OF INTEREST

The authors declare that they do not have any conflict of interest.

## ETHICAL STATEMENTS

This study was approved by the Ethical Committee of Ocean University of China (Approved protocol ID SPXY2019042001) and complied with the Guide for the Care and Use of Laboratory Animals (NIH, 8th Edition). Informed Consent: Written informed consent was obtained from all study participants.

## Supporting information

Table S1‐3

## Data Availability

The data that support the findings of this study are available in the supplementary material of this article. Table S1, Detailed information of up‐regulated genes in H5% mice; Table S2, Detailed information of down‐regulated genes in H5% mice; Table S3, The original data of body weight and fat rate of mice in both groups.
