# Supplementary material for: The mechanism exploration of the non‐colonic toxicity and obesity inhibition of food‐grade κ‐carrageenan by transcriptome
Source: Food Sci Nutr. 2021 Sep 28;9(11):6232–44. doi: 10.1002/fsn3.2581 (PMC8565199; doi:10.1002/fsn3.2581)
Supplement: Supplementary file 1 — Table S1‐3 [file FSN3-9-6232-s001.docx]

**Supplementary Material**

**Supplementary Table S1 The Fold Change and Annotation of Up-regulated Genes in H5% Mice**

| **Gene** | **FC(H5%/HFD)** | ***p*-value** | **KEGG Pathway** |  |  |
| --- | --- | --- | --- | --- | --- |
| Timp1 | 2.076 | 0.004159483 | HIF-1 signaling pathway |  |  |
| Sgca | 2.407 | 0.000101412 | Viral myocarditis |  |  |
| Folr1 | 2.007 | 0.047484631 | Antifolate resistance; Endocytosis |  |  |
| Kdelr3 | 2.357 | 0.00127066 | Vibrio cholerae infection |  |  |
| Cxcl14 | 2.188 | 0.000749096 | Cytokine-cytokine receptor interaction; |  |  |
| Clps | 3.009 | 0.011501958 | Fat digestion and absorption |  |  |
| Abcg8 | 3.162 | 0.009943742 | Cholesterol metabolism; ABC transporters; Bile secretion; Fat digestion and absorption |  |  |
| Slc16a3 | 2.138 | 0.016184364 | Central carbon metabolism in cancer | | |
| Des | 2.324 | 3.04253E-05 | Dilated cardiomyopathy (DCM); Arrhythmogenic right ventricular cardiomyopathy (ARVC); Hypertrophic cardiomyopathy (HCM) |  |  |
| Lct | 4.127 | 0.003411376 | Carbohydrate digestion and absorption; Galactose metabolism |  |  |
| Batf3 | 2.116 | 0.022802604 | PD-L1 expression and PD-1 checkpoint pathway in cancer | | |
| Lrp2 | 2.85 | 0.002499996 | Hedgehog signaling pathway; Thyroid hormone synthesis; Cholesterol metabolism | | |
| Chac1 | 2.003 | 0.042114904 | Glutathione metabolism | |  |
| Bdh2 | 2.029 | 0.002368582 | Synthesis and degradation of ketone bodies; Butanoate metabolism | | |
| Pla2g2f | 2.288 | 0.035844936 | Ether lipid metabolism; Glycerophospholipid metabolism; Arachidonic acid metabolism; Linoleic acid metabolism; Pancreatic secretion; |  |  |
| Hcrtr1 | 2.084 | 0.042631833 | Neuroactive ligand-receptor interaction | | |
| Areg | 2.002 | 0.000456235 | Colorectal cancer; Hippo signaling pathway; MAPK signaling pathway; ErbB signaling pathway; PI3K-Akt signaling pathway |  |  |
| Fgf15 | 12.877 | 0.024435024 | Regulation of actin cytoskeleton; Pathways in cancer; MAPK signaling pathway; Breast cancer; Gastric cancer; PI3K-Akt signaling pathway |  |  |
| Pla2g4c | 2.183 | 0.024089654 | Ether lipid metabolism; Inflammatory mediator regulation of TRP channels; MAPK signaling pathway; Phospholipase D signaling pathway | | |
| Gna15 | 2.084 | 0.022833499 | Chagas disease (American trypanosomiasis); Amoebiasis; Relaxin signaling pathway; Calcium signaling pathway |  |  |
| Nr0b2 | 33.585 | 0.015084073 | Bile secretion |  |  |
| Bdkrb1 | 2.774 | 0.03315994 | Regulation of actin cytoskeleton; Inflammatory mediator regulation of TRP channels; Pathways in cancer; Neuroactive ligand-receptor interaction; |  |  |
| Notum | 2.901 | 0.008101822 | Wnt signaling pathway |  |  |
| Vsig4 | 14.708 | 0.03398182 | Complement and coagulation cascades |  |  |
| Defb1 | 6.38 | 0.006901912 | ABC transporters; Staphylococcus aureus infection |  |  |
| Krt14 | 2.302 | 0.008278989 | Staphylococcus aureus infection; Estrogen signaling pathway |  |  |
| Dnah14 | 3.454 | 0.011899757 | Huntington disease | | |
| Cox7c-ps1 | 2.838 | 0.02234642 | Oxidative phosphorylation; Parkinson disease; Alzheimer disease; Huntington disease; Thermogenesis; Non-alcoholic fatty liver disease (NAFLD) |  |  |
| Gm10037 | 4.704 | 0.037669399 | Herpes simplex virus 1 infection |  |  |
| Actg2 | 2.28 | 1.31371E-06 | Vascular smooth muscle contraction |  |  |
| Mcpt4 | 3.31 | 0.046541601 | Renin-angiotensin system | | |
| Myl4 | 2.59 | 4.74323E-06 | Cardiac muscle contraction; Adrenergic signaling in cardiomyocytes; Apelin signaling pathway |  |  |
| Rpl21-ps8 | 22.63 | 0.002905946 | Ribosome |  |  |
| Gm10108 | 301.756 | 0.014950534 | Non-alcoholic fatty liver disease (NAFLD); Epstein-Barr virus infection; Colorectal cancer; Parkinson disease; Alzheimer disease; |  |  |
| Acat3 | 2.047 | 0.042935013 | Terpenoid backbone biosynthesis; Synthesis and degradation of ketone bodies; Fatty acid degradation; amino acid metabolism; Pyruvate metabolism |  |  |
| Gm14176 | 4.766 | 0.02190746 | MicroRNAs in cancer; RNA transport; Ubiquitin mediated proteolysis; NF-kappa B signaling pathway | | |
| mt-Atp6 | 2.402 | 0.001523644 | Parkinson disease; Alzheimer disease; Huntington disease; Thermogenesis; Oxidative phosphorylation | | |
| Sirpb1c | 6.075 | 0.04684413 | Osteoclast differentiation |  |  |
| Ighg1 | 2.208 | 0.031587246 | Natural killer cell mediated cytotoxicity; Dilated cardiomyopathy (DCM); NF-kappa B signaling pathway; Primary immunodeficiency | | |
| Ighv14-4 | 2.266 | 0.032399038 | NF-kappa B signaling pathway; Primary immunodeficiency; Intestinal immune network for IgA production; PI3K-Akt signaling pathway |  |  |
| Ighv7-4 | 3.974 | 0.019868104 | Phospholipase D signaling pathway; Autoimmune thyroid disease; Hematopoietic cell lineage; Phagosome; Fc epsilon RI signaling pathway; |  |  |
| Rpl23a-ps3 | 26.084 | 0.014706986 | Ribosome |  |  |
| Ackr4 | 2.126 | 3.67853E-09 | Cytokine-cytokine receptor interaction;Viral protein interaction with cytokine and cytokine receptor | | |
| Gm9385 | 22.737 | 0.014719688 | Ribosome | | |
| Ndufs5-ps | 11.981 | 0.034793373 | Non-alcoholic fatty liver disease (NAFLD); Parkinson disease; Alzheimer disease; Huntington disease; Thermogenesis; Oxidative phosphorylation |  |  |
| Gm11249 | 5.493 | 0.037639987 | Ribosome |  |  |
| Gm11662 | 18.478 | 0.016204386 | Ribosome |  |  |
| Hoxa11os | 2.008 | 0.023334791 | Transcriptional misregulation in cancer |  |  |
| Gm5244 | 2.321 | 0.005566243 | Ribosome |  |  |
| Ighv1-36 | 39.034 | 0.002174052 | Natural killer cell mediated cytotoxicity; Primary immunodeficiency; Intestinal immune network for IgA production; PI3K-Akt signaling pathway |  |  |
| n-R5s111 | 29.011 | 0.025854151 | Basal transcription factors; Ubiquitin mediated proteolysis; Primary immunodeficiency |  |  |
| Ighv1-84 | 24.407 | 0.038516333 | Fc epsilon RI signaling pathway; B cell receptor signaling pathway; NF-kappa B signaling pathway; Intestinal immune network for IgA production; |  |  |
| Ighv5-4 | 2.673 | 0.035372446 | Hematopoietic cell lineage; Phagosome; NF-kappa B signaling pathway; Primary immunodeficiency; Intestinal immune network for IgA production; |  |  |
| Gm5451 | 17.169 | 0.035725041 | Ribosome |  |  |
| n-R5s134 | 25.411 | 0.033732164 | Basal transcription factors; Ubiquitin mediated proteolysis; Primary immunodeficiency | | |
| Gm5559 | 2.024 | 0.021114202 | Glycolysis / Gluconeogenesis; Alzheimer disease; Pathogenic Escherichia coli infection; Salmonella infection; HIF-1 signaling pathway | | |
| Gm26885 | 2.282 | 0.041095713 | Pathways in cancer; Jak-STAT signaling pathway; Acute myeloid leukemia; AGE-RAGE signaling pathway in diabetic complications |  |  |
| Gm5619 | 6.307 | 0.037638399 | Ribosome | | |
| Gm29216 | 43.571 | 0.001382039 | Oxidative phosphorylation; Cardiac muscle contraction; Parkinson disease; Alzheimer disease; Huntington disease; Thermogenesis |  |  |
| Gm44985 | 2.225 | 0.007561399 | Cell cycle; Oocyte meiosis; FoxO signaling pathway; Progesterone-mediated oocyte maturation |  |  |
| Gpx4-ps2 | 2.411 | 0.037331687 | Glutathione metabolism; Ferroptosis |  |  |
| Gm7143 | 4.114 | 0.048117098 | Ribosome |  |  |
| Gm7633 | 15.191 | 0.040867845 | Ribosome |  |  |
| Gm49392 | 3.588 | 0.037667767 | Proteoglycans in cancer; Rheumatoid arthritis; Fluid shear stress and atherosclerosis; Phagosome; Apoptosis; Antigen processing and presentation; |  |  |
| Gm49591 | 9.163 | 0.032468662 | Cell cycle; Huntington disease; Chronic myeloid leukemia; Transcriptional misregulation in cancer; Thyroid hormone signaling pathway |  |  |
| Gm49804 | 2.682 | 0.0025524 | Ribosome |  |  |

Only the genes that can be annotated in the KEGG PATHWAY database are shown, and only the major pathways that the genes are involved in are shown, FC≥2 & *p*<0.05.

**Supplementary Table S2 The Fold Change and Annotation of Down-regulated Genes in H5% Mice**

| Gene | FC(H5%/HFD) | *p*-value | KEGG Pathway |
| --- | --- | --- | --- |
| Zim1 | 0.378 | 0.038686 | Herpes simplex virus 1 infection |
| Cd36 | 0.433 | 0.000317 | PPAR signaling pathway; Adipocytokine signaling pathway; Cholesterol metabolism; Fat digestion and absorption; AMPK signaling pathway |
| Slc1a3 | 0.492 | 0.046362 | Huntington disease; Glutamatergic synapse; Synaptic vesicle cycle |
| Trpv1 | 0.167 | 0.048948 | Neuroactive ligand-receptor interaction; Inflammatory mediator regulation of TRP channels |
| Acsl1 | 0.424 | 0.000876 | Fatty acid degradation; Adipocytokine signaling pathway; PPAR signaling pathway; Thermogenesis;Peroxisome; Ferroptosis; Fatty acid biosynthesis |
| Sycp3 | 0.278 | 0.044573 | Homologous recombination |
| Wif1 | 0.468 | 0.020043 | Wnt signaling pathway |
| Slc36a2 | 0.405 | 0.032655 | Protein digestion and absorption |
| Cpb2 | 0.24 | 0.001602 | Pancreatic secretion; Protein digestion and absorption; Complement and coagulation cascades |
| Ms4a1 | 0.452 | 0.002903 | Hematopoietic cell lineage |
| Cr2 | 0.399 | 0.004938 | Hematopoietic cell lineage; B cell receptor signaling pathway; Epstein-Barr virus infection; Complement and coagulation cascades |
| Pfkfb3 | 0.495 | 0.002253 | Fructose and mannose metabolism; AMPK signaling pathway; HIF-1 signaling pathway |
| Mme | 0.472 | 0.016365 | Hematopoietic cell lineage; Protein digestion and absorption; Alzheimer disease; Renin-angiotensin system |
| Aqp7 | 0.435 | 0.000368 | Regulation of lipolysis in adipocytes; PPAR signaling pathway |
| Pla2g2c | 0.494 | 0.043857 | Arachidonic acid metabolism; Linoleic acid metabolism; Fat digestion and absorption; Pancreatic secretion; alpha-Linolenic acid metabolism |
| Per3 | 0.499 | 4.41E-05 | Transcriptional misregulation in cancer; Circadian entrainment; Circadian rhythm; Acute myeloid leukemia |
| Gipr | 0.387 | 0.013082 | cAMP signaling pathway; Neuroactive ligand-receptor interaction |
| Adrb3 | 0.444 | 0.015099 | Thermogenesis; Salivary secretion; Regulation of lipolysis in adipocytes; Calcium signaling pathway; cGMP-PKG signaling pathway |
| Acss3 | 0.309 | 0.003191 | Propanoate metabolism |
| Stag3 | 0.158 | 0.015996 | Oocyte meiosis |
| Scd1 | 0.314 | 0.041031 | PPAR signaling pathway; AMPK signaling pathway; Biosynthesis of unsaturated fatty acids |
| Serpinf2 | 0.344 | 0.007371 | Complement and coagulation cascades |
| Iapp | 0.265 | 0.043386 | Neuroactive ligand-receptor interaction; Maturity onset diabetes of the young |
| Mc2r | 0.281 | 0.004573 | cAMP signaling pathway; Neuroactive ligand-receptor interaction; Aldosterone synthesis and secretion; Cortisol synthesis and secretion; Cushing syndrome |
| Irs3 | 0.432 | 0.042489 | Adipocytokine signaling pathway; Regulation of lipolysis in adipocytes; Insulin signaling pathway; AMPK signaling pathway |
| Hcar1 | 0.467 | 0.029127 | cAMP signaling pathway |
| Sec31b | 0.433 | 0.018941 | Protein processing in endoplasmic reticulum |
| F730043M19Rik | 0.499 | 0.043476 | Insulin signaling pathway; Longevity regulating pathway; EGFR tyrosine kinase inhibitor resistance; PI3K-Akt signaling pathway; HIF-1 signaling pathway |
| Rxfp2 | 0.066 | 0.044569 | Neuroactive ligand-receptor interaction; Relaxin signaling pathway |
| H2-T24 | 0.436 | 0.047862 | Human T-cell leukemia virus 1 infection; Autoimmune thyroid disease; Type I diabetes mellitus; Phagosome; Antigen processing and presentation |
| Abcd2 | 0.417 | 0.004007 | Peroxisome; ABC transporters |
| Chst3 | 0.469 | 0.040651 | Glycosaminoglycan biosynthesis - chondroitin sulfate / dermatan sulfate |
| Lep | 0.179 | 0.002374 | Adipocytokine signaling pathway; Cytokine-cytokine receptor interaction; Non-alcoholic fatty liver disease (NAFLD); AMPK signaling pathway |
| H2ac10 | 0.075 | 0.0411 | Systemic lupus erythematosus; Alcoholism; Necroptosis |
| Fabp4 | 0.473 | 0.001182 | Regulation of lipolysis in adipocytes; PPAR signaling pathway |
| Nupl1 | 0.042 | 0.002387 | RNA transport |
| Gm10143 | 0.052 | 0.025546 | Base excision repair; DNA replication; Non-homologous end-joining |
| Serpina1a | 0.094 | 0.010656 | Complement and coagulation cascades |
| Zfp951 | 0.448 | 0.00018 | Herpes simplex virus 1 infection |
| Amy1 | 0.448 | 0.00901 | Pancreatic secretion; Carbohydrate digestion and absorption; Starch and sucrose metabolism |
| Zfp558 | 0.386 | 0.039156 | Herpes simplex virus 1 infection |
| Il22 | 0.084 | 0.014366 | Th17 cell differentiation; Jak-STAT signaling pathway; Inflammatory bowel disease (IBD); Cytokine-cytokine receptor interaction |
| Ighv6-3 | 0.251 | 0.026696 | Leishmaniasis; Natural killer cell mediated cytotoxicity; Amoebiasis; African trypanosomiasis; infection; pathway; Primary immunodeficiency; |
| Sms-ps | 0.097 | 6.53E-05 | Glutathione metabolism; Cysteine and methionine metabolism; Arginine and proline metabolism |
| Gm14150 | 0.111 | 0.040982 | Gap junction; Alzheimer disease; Huntington disease; Pathogenic Escherichia coli infection; Phagosome; Apoptosis; Tight junction |
| Gm16233 | 0.186 | 0.03126 | Pancreatic secretion; Renin secretion |
| Gm12905 | 0.128 | 0.040134 | Shigellosis; mTOR signaling pathway; Autophagy - animal |
| B130055M24Rik | 0.316 | 0.013083 | Herpes simplex virus 1 infection |
| 4931428L18Rik | 0.112 | 0.031412 | Herpes simplex virus 1 infection; Spliceosome; RNA transport; mRNA surveillance pathway |
| Gm7162 | 0.361 | 0.047423 | RNA transport |
| Ighv1-76 | 0.129 | 0.000145 | Natural killer cell mediated cytotoxicity; Phospholipase D signaling pathway; Autoimmune thyroid disease; Pathogenic Escherichia coli infection; |
| Ighv1-80 | 0.369 | 0.007993 | NF-kappa B signaling pathway; Primary immunodeficiency; Allograft rejection; Intestinal immune network for IgA production;PI3K-Akt signaling pathway |
| Ighv1-78 | 0.29 | 0.016705 | Leishmaniasis; Natural killer cell mediated cytotoxicity; Amoebiasis; Asthma; Epstein-Barr virus infection; African trypanosomiasis; |
| Gm13137 | 0.434 | 0.035235 | Ribosome |
| Gm36936 | 0.211 | 0.007492 | Epstein-Barr virus infection; Ubiquitin mediated proteolysis; Endocytosis; Tight junction |
| Gm37988 | 0.104 | 0.022166 | Glycerophospholipid metabolism; Choline metabolism in cancer |
| Gm45380 | 0.08 | 0.012786 | Ribosome |
| Gm47766 | 0.119 | 0.037099 | Regulation of actin cytoskeleton; Systemic lupus erythematosus; Adherens junction; Leukocyte transendothelial migration; Tight junction; |

Only the genes that can be annotated in the KEGG PATHWAY database are shown, and only the major pathways that the genes are involved in are shown, FC≤0.5 & *p*<0.05.

**Supplementary Table S3 The Body-weight and Fat-rate of mice**

| Phenotypes  Groups | Body weight (g) | Body rat rate (%) |
| --- | --- | --- |
| HFD-1 | 37.68 | 11.78 |
| HFD-2 | 38.31 | 12.28 |
| HFD-3 | 30.17 | 4.73 |
| HFD-4 | 32.53 | 10.01 |
| H5%-1 | 29.23 | 3.84 |
| H5%-2 | 31 | 4.58 |
| H5%-3 | 29.40 | 5.47 |
| H5%-4 | 25.08 | 1.73 |
